# Construction of Digital Management Model of Personnel Archives Information Based on the Concept of Industry 4.0

**DOI:** 10.1155/2022/9328566

**Published:** 2022-06-07

**Authors:** Jiaonan Han

**Affiliations:** Human Resources Business Consulting Department, Kunlun Digital Technology Co., Ltd., Beijing 100007, China

## Abstract

In order to improve the effect of personnel archives information management under the background of Industry 4.0, this paper conducts in-depth research on retrieval technology. Aiming at the shortcomings and deficiencies of the existing sorting methods, this paper introduces the basic principle of information retrieval, the retrieval process, and retrieval model and proposes a relevance ranking algorithm based on the user vector calculation method and the record vector calculation method. The algorithm solves the query structure sorting problem of information retrieval, optimizes the retrieval process, and improves the efficiency of information acquisition. Under the guidance of the concept of Industry 4.0, this paper constructs the digital management model of personnel archives information. The experimental results show that the digital management model of personnel archives proposed in this paper has certain effects.

## 1. Introduction

Industry 4.0 advocates the management of personnel archives in a digital form, promotes the intelligent protection of personnel archives management, and improves the efficiency of personnel archives management.

Personnel archives are personal archives in the form of words, images, and sounds, which are formed in the process of personnel management and are recognized by the organization, including personal information, personal experience, performance of morality, and talent. Personnel archives are an important part of national archives management. The so-called digital construction of personnel archives is the process of forming a systematic and structured personnel archives information database from traditional paper media, audio and video archives, and scattered electronic personnel archives under the support of database technology and data compression and scanning technology. The systematic and structured personnel archives formed by the digitalization of personnel archives exist in a digital form with the support of database technology and are called digital archives. With the support of computer technology and network technology, digital information is more conducive to the sharing of resources and effectively improves the efficiency of resource utilization. In terms of work content, the digital construction of personnel archives mainly includes two parts. One is to transform traditional archives into digital archives through various technologies such as data scanning, data compression, and database; the other is to transform various scattered archives into digital archives. Personal electronic data, personal electronic data generated by different organizations, are systematically organized to form systematic archives. With the support of computer technology and network technology, digital cognition archives can provide users with functions such as network-based archives search, access, and utilization and can be connected with various current office automation systems. The use of accuracy is extremely important.

In the traditional personnel archives management mode, the search, use, analysis, and transmission of archives need to be done manually. The manual work is not only inefficient, but also prone to various errors. Through digital construction, traditional paper archives are transformed into digital information, and the work efficiency can be greatly improved through computer search, identification, analysis, application, and transmission, and its accuracy can be effectively guaranteed. In addition, through database technology and network technology, digital archives can also realize the integrated service of application, inquiry, and utilization on the network and further expand the application scope of incoming archives. This also promotes the innovation of the use of archives and makes the personnel archives play a greater role and value for the society.

With the development of the times, the importance of talents is self-evident, and the digital construction of personnel archives can effectively promote the development and application of talent information. Personnel archives under the traditional mode can often only play the role of postmortem investigation. Because of the low efficiency of manual operations, it is extremely difficult to search for talents from a large number of personnel archives according to certain requirements. Moreover, under the promotion of the digital construction of personnel archives, the massive data in the personnel archives can be automatically retrieved through the computer, so as to quickly provide the talent data that meets the requirements. In addition, it can achieve classification, sorting, screening, selection, and other work according to needs, so as to provide greater help for the leadership of personnel decision-making. From this perspective, the digital construction of personnel archives is conducive to the selection, cultivation, and retention of talents.

There is also a wealth of information in personnel archives, so their security and confidentiality are paramount. However, in the traditional archives management mode, manual operations will inevitably have various oversights. Whether it is in the collection and arrangement, or in all aspects of storage and transmission, there may be data loss or leakage, which will bring adverse effects. For example, the frequent occurrence of archives fraud in recent years shows that there are large loopholes in the traditional archives management model. The digital construction of personnel archives not only improves the utilization efficiency of archives, but also better protects the archives themselves. At the same time, with the support of professional technology, it is almost impossible to modify and exchange archives, which also ensures the security and confidentiality of archives.

## 2. Related Work

Pay attention to and do a good job in the collection, sorting, and auditing of archives. In order to further improve the digital management level of personnel archives, archives managers should start from the three links of archives collection, archives arrangement, and archives review and promote the continuous development of personnel archives management in the direction of digitization [1]. In this process, the archives management personnel should simplify the archives verification process on the basis of ensuring the authenticity and effectiveness of the information data [2]. According to certain standards, scientific classification of archives information should be carried out, and the classified information should be counted and marked by serial number, so as to ensure the conciseness and clarity of archives information, so that relevant personnel can quickly find it in the first time. And call the archives information you need [3]. According to the actual needs of personnel archives management, the archives management personnel shall verify the personnel archives and check the archives information one by one to ensure the comprehensiveness, integrity, and authenticity of all archives information. If it is found that the format of the archives information filled in is not rigorous and nonstandard, it is necessary to reenter and modify the information to ensure the standardization and standardization of the fill-in format of all archives information [4]. The archives management personnel should also regularly supervise and review the personnel archives, find and solve the problems existing in the personnel archives in time, and create favorable conditions for further improving the management level of the personnel archives [5]. Establish and improve the personnel archives database. In order to realize the digital management of personnel archives, archive managers should also pay attention to the establishment and improvement of the database of personnel archives [6]. According to the actual management status of personnel archives, the archives management personnel should establish and improve the archives information database to ensure the standardization and standardization of the contents of the database. Ensure that social security, provident fund, and other information data are accurately stored in the information database [7]. It provides an important platform support for promoting the effective connection between paper materials and electronic materials in the later period and improving the authenticity and integrity of archive information. Finally, it is necessary to make full use of and give full play to the application advantages of digital technology to ensure that archive information can be entered and stored accurately, reliably, and safely [8]. Strengthen archive information security management. In order to promote the development of the digital management of personnel archives in a standard, normative, and scientific direction, the archives management personnel should increase the management of archives information security. In this process, first of all, it is necessary to strictly follow the relevant standards and requirements, starting from the entry, review, and storage of archive information, to improve the standardization of the archive operation process [9]. It is necessary to separate the storage place of personnel archives from the office of personnel archives and complete the establishment of the archives. It is strictly forbidden for outsiders to enter the archives without authorization to search and transfer personnel archives. Secondly, it is also mandatory to require archives managers and digital construction personnel to sign a confidentiality agreement for archives information to avoid loss or leakage of archives information due to human factors, so as to improve the security of archives information [10]. Adopt the method of closed system interface, and prohibit the access of external network and the establishment of wireless network. Finally, it is necessary to regularly maintain and update the archives information management system to improve the security of the system's operation and avoid the intrusion and tampering of archives information due to network viruses, hackers, and criminals [11].

Build perfect and unified information construction standards. In order to further improve the level of talent service informatization construction, archives managers should pay attention to the construction and improvement of informatization construction standards to ensure the uniformity and standardization of informatization construction standards. In this process, first of all, according to the actual construction needs of talent services, we should uniformly formulate and improve information technology application standards and effectively combine the rest of the talent informatization management standards to achieve the purpose of improving the level of talent informatization services [12]. It is necessary to strengthen the application of the talent information management system. In the context of the application of the system, by using the network, the sharing and utilization of information resources can be greatly improved, and a solid foundation will be laid for further improving the efficiency and effect of talent services [13]. It is necessary to uniformly use the talent service informatization construction technology application platform to ensure that different departments can effectively collaborate under the condition of clear division of labor and avoid the problem of repeated collection and management of resources [14]. Increase the training of information technology talents. Information technology talents play an important role in realizing the informatization construction of talent services. Therefore, archives managers should strengthen the training of information technology personnel and continuously improve the professional ability and professional quality of information technology personnel [15]. According to the development needs of talent services, a number of informatization construction groups are established, so that these groups can fully understand and grasp the needs of talent services with the cooperation and cooperation of other departments. Finally, an information system that meets the development needs of talent services is constructed to promote the continuous development of talent services in the direction of informatization and intelligence [16]. Formation of professional information technology institutions: in order to realize the informatization construction of talent service, archives managers should also pay attention to the establishment of professional information technology institutions and improve the efficiency and effect of talent service work by leveraging the role and strength of this institution [17]. In this process, first of all, on the basis of comprehensive consideration of the needs of talent service construction, the world's top first-class talents should be hired to participate in the formation of information technology professional institutions, so as to achieve the purpose of updating and improving the functions of the talent information system. Secondly, it is necessary to uniformly formulate talent management norms to ensure that relevant mechanisms and systems can play a real guiding and supervisory role in the construction of talent service informatization [18].

## 3. Personnel Information Digital Management Algorithm

Information retrieval refers to the process and technology of organizing information in a certain way and finding relevant information according to the needs of information users. For a text retrieval system, it mainly includes three parts: index generation, query processing, and document retrieval.

The simplest processing method of the retrieval system is to query the keywords and provide the result document to the user. This method does not process the result set of the query, and its working method is relatively simple. It does not take into account the reliability of the user at all, and the user may need to view the last result to find the document they need. Therefore, the retrieval system needs to sort the results of the query by relevance and provide feedback regarding the documents with the highest reliability to the user. Furthermore, we utilize an information retrieval model to compute the relevance of user queries and results. Information retrieval model is one of the core contents in information retrieval, including the following three classic models:

The Boolean model is the simplest retrieval model, and it is implemented based on set theory. In this model, each document is composed of a set of words that make up the document, and the user's query conditions are also composed of a set of words. For example, the Boolean model of a document *k* is *D*_*k*_=(*T*_1_, *T*_2,_*T*_3_,…, *T*_*n*_), and the query condition for the user is *i*, *S*_*i*_=(*T*_1_OPT_3_) AND (*T*_2_ORT_4_). In this way, through a simple Boolean algebra processing method, the corresponding documents are queried according to the user's query conditions. The advantage of Boolean query is that the processing method is simple and easy, but the disadvantage is that the retrieved results are not ordered, the retrieval strategy is single, and the precision rate is low.

**Figure 1:**
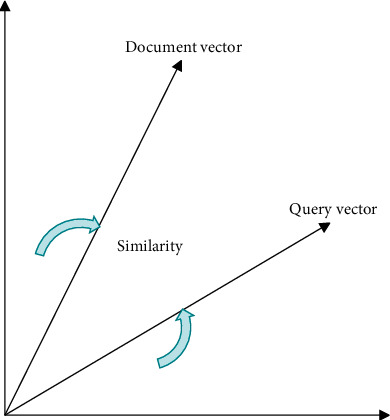
Schematic diagram of the vector model.

The vector model regards each document as a vector of several words that make it up, each word has its own weight, and the user's query conditions are also processed by such vectors. In the query process, the similarity between the user's query condition and the document is judged by the weight. Its processing model mainly includes three parts. First, the words in the document are represented by vectors. Second, the words in the query are represented by vectors. Finally, a method is found to calculate the similarity between the document vector and the query vector, and its processing model is shown in Figure 1.

The document vector representation is shown in formula (1), and the query vector representation is shown in formula (2).

(1) $$\begin{array}{c} D_{j}\left(W_{1j},W_{2j},\ldots ,W_{nj}\right). \end{array}$$

In the formula, *n* represents the number of feature words.

*W*_*ij*_ represents the weight representation of feature word *i* in *D*_*j*_.

(2) $$\begin{array}{c} Q\left(W_{1q},W_{2q},\ldots ,W_{nq}\right). \end{array}$$

In the formula, *W*_*iq*_ represents the weight representation of feature word *i* in *Q*.

The calculation method of the weight includes Boolean weight and TF weight, where the Boolean weight is *W*_*ij*_ = 1 or *W*_*ij*_ = 0, and the TF weight is the total number of keywords in the archive, *W*_*ij*_ = *TF*_*ij*_, or the normalized TF value. The three calculation methods of the weight are shown in the following formula:

(3) $$\begin{array}{c} \frac{TF_{i}}{\text{Max}TF_{i}}\text{OR}0.5+0.5\times \frac{TF_{i}}{\text{Max}TF}\text{OR}\frac{TF_{i}}{\sqrt{\sum_{i=1}^{t}TF_{i}^{2}}}. \end{array}$$

DF is the document frequency of the keyword, and IDF is the inverse document frequency of the keyword, IDF=log*N*/*DF*. In the vector space model *W*_*ij*_=*TF*_*ij*_ × IDF_*ij*_, the cosine function of the document vector and the query vector is used to calculate the similarity, as shown in the following formula:

(4) $$\begin{array}{c} sim\left(d_{j},q\right)=\frac{d_{j}\cdot q}{\left|d_{j}\right|\times \left|q\right|}\\ =\frac{\sum_{i=1}^{t}W_{ij}\times W_{iq}}{\sqrt{\sum_{i=1}^{t}W_{ij}^{2}\times \sum_{i=1}^{t}W_{iq}^{2}}}. \end{array}$$

The advantage of vector query is that the queried documents can be sorted according to their relevance, and the precision rate is high.

The probability model is derived from the principle of probability sorting. The basic idea is to calculate the relevant document probability *R* and the irrelevant document probability NR for the query request. *R* is initially a guessed value. Then, the similarity between the document *d*_*j*_ and the query *q* is shown in the following formula:

(5) $$\begin{array}{c} \text{sim}\left(d_{j},q\right)=\frac{p\left(R|d_{j}\right)}{p\left(NR|d_{j}\right)}. \end{array}$$

The Bayesian formula is shown in formula (6), and formula (5) is processed according to this formula, and the result is obtained, as shown in formula (7).

(6) $$\begin{array}{c} P\left(A|B\right)=\frac{P\left(B|A\right)\times P\left(A\right)}{P\left(B\right)}, \end{array}$$

(7) $$\begin{array}{c} \text{sim}\left(d_{j},q\right)=\frac{P\left(d_{j}|R\right)\times P\left(R\right)}{P\left(d_{j}|NR\right)\times P\left(NR\right)}. \end{array}$$

In the probabilistic model processing process, a probabilistic model is first established for the query conditions of the document and the user, and then, the query result is obtained by calculating the similarity between the two. The advantage of the probability model is that the model is self-adaptive, and the precision rate will increase with the increase of the number of queries. The disadvantage is that there are many human factors in the processing process, the process is complicated, and a certain process is required to improve the precision rate.

**Figure 2:**
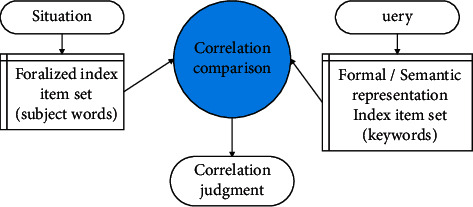
Basic pattern of system dependencies.

System correlation is the matching relationship between the document and the query detected by the information retrieval system from the document set for the user's query. It is believed that relevance is mainly affected by document characteristics and operations. These inherent characteristics and operations mainly include classification table, subject thesaurus, index, semantic and grammatical description of vocabulary, document organization, and analysis and retrieval strategy of retrieval questions. The basic pattern of system dependencies is shown in Figure 2.

The essence of system relevance is one-sided, and it only looks at the importance of the system in the query process and does not consider the user's perception.

**Figure 3:**
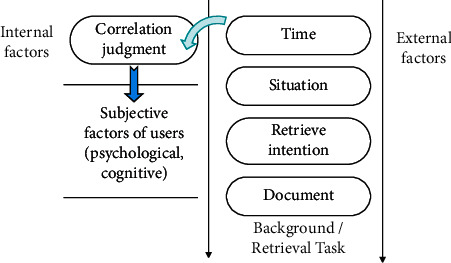
Basic patterns of user relevance.

User relevance is the matching relationship between the query issued by the user and the user's real information needs. With the development of information retrieval technology, the amount of data in the retrieval process is increasing, and user perception plays an extremely important role in the process of information retrieval. In the process of querying, the user's psychology undergoes a change process, which is affected by the user's personal psychological environment and the external environment. The basic mode of user correlation is shown in Figure 3.

User-oriented relevance can better reflect the reliability of retrieval results than system-oriented relevance. However, due to the influence of internal factors of users, the same retrieval process may produce different results in different contexts and cannot be quantified. The current mainstream quantitative correlation evaluation mainly includes recall rate, precision rate, and the precision of the first *n* items. Since the user only pays attention to the first few results of the query result, the similarity of the first *n* items is an evaluation index.

Relevance is the quantification of relevance, and many retrieval systems use the relevance ranking method to optimize the result set, so that the most relevant records are ranked first. Traditional relevance ranking models include the following.

### 3.1. Query Related Models

The model is a model that ranks based on content relevance, such as traditional Boolean models, vector models, and probabilistic models. The Boolean model can only judge whether the correlation exists and cannot be quantified, while the vector model can be quantified, but the effect of probability sorting is better, such as the famous language model BM25 model.

The BM25 model is shown in the following formula:

(8) $$\begin{array}{c} BM25\left(d,q\right)=\sum_{i=1}^{M}\frac{\text{IDF}\left(t_{i}\right)\cdot TF\left(t_{i},d\right)\cdot \left(k_{I}+1\right)}{TF\left(t_{i},\;d\right)+K_{I}\cdot \left(1-b+b\cdot \text{LEN}\left(d\right)/\text{avdl}\right)}. \end{array}$$

In the formula, TF(*t*,*d*) is the term frequency of term *t* in document *d*, LEN(*d*) is the number of terms in document *d*, and avdl is the average length of documents in the text collection, IDF(*t*) is the IDF weight of the entry *t*, and *t*_*i*_ is the background language model.

The language model format is shown in the following formula:

(9) $$\begin{array}{c} p\left(t_{i}|d\right)=\left(1-\varphi \right)\frac{TF\left(t_{i},d\right)}{\text{LEN}\left(d\right)}+\varphi p\left(t_{i}|C\right). \end{array}$$

In the formula, K1 and *b* are parameters, *p*(*t*_*i*_|*C*) is the entry, and *φ* ∈ [0,1] is the smoothing factor.

The query-independent model uses the link method to calculate the relevance, such as the Hyperlink algorithm, the HITS algorithm, the TrustRank algorithm, and the PageRank algorithm, among which the most famous algorithm is the PageRank algorithm, and the relevance is shown in the following formula:

(10) $$\begin{array}{c} PR\left(d_{u}\right)=\sum_{d_{v}\in B_{u}}\frac{PR\left(d_{v}\right)}{U\left(d_{v}\right)}. \end{array}$$

*U*(*d*_v_) means that a smoothing factor is added to the actual application of the number of out-chains of *d*_v_, which is shown in the following formula:

(11) $$\begin{array}{c} PR\left(d_{u}\right)=\alpha \times \sum_{d_{v}\in B_{u}}\frac{PR\left(d_{v}\right)}{U\left(d_{v}\right)}+\frac{1-\alpha }{N}. \end{array}$$

In the formula, *N* is the number of all pages, and *α* is the damping coefficient.

If it is assumed that *n* keywords are obtained after the query string input by the user is segmented, the order of the keywords indicates their importance, and they are given different weights according to their importance. We define the keyword weight of keyword *l* as shown in the following formula:

(12) $$\begin{array}{c} W_{zy}\left(l\right)=\frac{n-\left(l-1\right)}{\sum_{i=1}^{n}\left[n-\left(i=1\right)\right]}\;i,l\in \left[1,n\right]\;n\in z\text{ AND }n\geq 1. \end{array}$$

Among them, *W*_*zy*_(*l*) is the weight of the *l*-th keyword, *n* is the number of keywords, and *i* is the summation variable.

The number of user queries for a certain keyword reflects the popularity of the word, which depicts the importance of the keyword in work. The query semantics expressed by keywords with high popularity are more concerned by users. The more times a keyword is queried, the more important it is in the minds of users. Therefore, this algorithm considers the historical query times of keywords.

We assume that the database has been queried for *m* times, and there are *n* query keywords. The number of times the first keyword is queried is *f*1, the number of times the second keyword is queried is *f*2,…, and the number of times the nth keyword is queried is *f*_*n*_. The weight of the historical query times of the lth keyword is defined as shown in the following formula:

(13) $$\begin{array}{c} W_{ls}\left(l\right)=\frac{f_{l}}{\;m},\;l\in \left[1,n\right]. \end{array}$$

Among them, *W*_*ls*_(*l*) is the weight of the historical query times of the *l*-th keyword, *f*_1_ is the number of times the *l*-th keyword is queried, *m* is the total number of queries to the database, and *n* is the number of keywords.

The user vector is calculated by using the weight values *W*_*zy*_ and *W*_*ls*_, which is represented by *Q*, and the definition of *Q* is shown in the following formula:

(14) $$\begin{array}{c} Q_{l}=C_{1}W_{zy}\left(l\right)+C_{2}\omega W_{ls}\left(l\right)\\ =C_{1}\frac{n-\left(l-1\right)}{\sum_{i=1}^{n}\left[n-\left(i-1\right)\right]}+C_{2}\frac{\omega f_{l}}{m},\;i,l\in \left[1,n\right]\;C_{1}+C_{2}=1. \end{array}$$

Among them, *Q*_*l*_ is the *l*^th^ value of the user vector, *W*_*zy*_(*l*) is the weight of the *l*-th keyword, *W*_*ls*_(*l*) is the weight of the historical query times of the *l*^th^ keyword, and *f*_*l*_ is the *l*-th keyword being queried number of times. At the same time, *m* is the total number of queries to the database, *n* is the number of keywords, C1, C2, *ω* are adjustment constants, and *i* is a summation variable.

In formula (14), *ω*, C1, and C2 are all constants, and *ω* is a value for adjusting the weight of the historical query times of keywords according to the test results. Because if the value of *f*_*l*_/*m* is too small, its role in the calculation formula of the query vector is very small and can be ignored. Then, the impact of the number of historical queries on the relevance is not reflected. Therefore, its weight value should be adjusted according to the test results. The sum of C1 and C2 is 1. It represents the difference between the importance of keywords and the number of historical queries in the minds of users. Usually, the importance of keywords is more important than the number of historical queries, so we set C1 > C2.

System correlation is the comparison between the feature information inherent in the document itself and the feature information inherent in the query expression submitted by the user, including correlation based on similarity of vocabulary selection, correlation based on similarity of grammatical structure, correlation based on word frequency, and correlation based on probability, a probability-based correlation. Through analysis, it is found that the system correlation is related to two factors, so the corresponding vectors are recorded considering two factors.

The weight of the number of times a keyword appears in each attribute is represented by *W*_*cs*_.

The number of times a keyword appears is also called word frequency. When other factors are the same, the more the times a keyword appears in a record, the higher the relevance of the record to the user's query is.

There are *n* keywords, each of which is represented as *x*_1_, *x*_2_,…, *x*_*n*_.

We set a total of *δ* records to be found, and each record is represented as *r*_1_, *r*_2_,…, *r*_*δ*_.

There are *μ* fields, each of which is represented as *f*_1_, *f*_2_,…, *f*_*μ*_.

The number of occurrences of the lth keyword in the first attribute to the *μ*-th attribute of the first record is defined as shown in the following formula:

(15) $$\begin{array}{c} \lambda_{x_{l}r_{l}f_{1}},\lambda_{x_{l}r_{l}f_{2}},\ldots ,\lambda_{x_{l}r_{l}f_{\mu }}. \end{array}$$

The number of occurrences of each keyword in the *δ*-th record is defined as shown in the following formula:

(16) $$\begin{array}{c} \left[\begin{array}{cccc} \lambda_{x_{1}r_{\delta }f_{1}} & \lambda_{x_{2}r_{\delta }f_{1}} & \cdots & \lambda_{x_{n}r_{\delta }f_{1}}\\ \lambda_{x_{1}r_{\delta }f_{2}} & \lambda_{x_{2}r_{\delta }f_{2}} & \cdots & \lambda_{x_{n}r_{\delta }f_{2}}\\ \vdots & \vdots & \vdots \\ \lambda_{x_{1}r_{\delta }f_{\mu }} & \lambda_{x_{2}r_{\delta }f_{\mu }} & \cdots & \lambda_{x_{n}r_{\delta }f_{\mu }} \end{array}\right]. \end{array}$$

The total number of times that the first keyword appears in the first record is defined as shown in the following formula:

(17) $$\begin{array}{c} \lambda_{x_{I}r_{l}f_{I}}+\lambda_{x_{l}r_{l}f_{2}}+\cdots +\lambda_{x_{I}r_{l}f_{\mu }}. \end{array}$$

The weight of the number of occurrences of the *i*-th keyword in the *j*-th field of the *γ* record is defined as shown in the following formula:

(18) $$\begin{array}{c} W_{cs}\left(i,\gamma ,j\right)=\frac{\lambda_{x_{i}\gamma \prime \gamma_{j}}}{\sum_{j=1}^{\mu }\lambda_{x_{i}r_{\gamma }f_{j}}},\;i\in \left[1,n\right]\;j\in \left[1,\mu \right]\;\gamma \in \left[1,\delta \right]. \end{array}$$

Among them, *W*_*CS*_(*i*, *γ*, *j*) is the weight of the number of occurrences of the *i*-th keyword in the *j*-th field of the *γ*-th record, *λ*_*xjrγfj*_ is the number of occurrences of the *i*-th keyword in the *j*-th field of the *γ*-th record, and ∑_*j*=1_^*μ*^*λ*_*X*_*i*_*r*_*γ*_*f*_*j*__ is the number of times that the *i*-th keyword appears in all fields of the *γ*-th record. Meanwhile, *j* is the summation variable, and *μ* is the weight of the position where the field number keyword appears, which is represented by *W*_wz_.

Typically, a database will have several tables to store data. In each table in the database, there are many fields. Keywords appear in different fields, and their importance is different. For example, appearing in the title is more important than appearing in the main text, and appearing in the title proper is more important than appearing in the subtitle and footnotes. Therefore, we need to further consider which field the keyword appears in. If it is assumed that there are *μ* fields in total, the position weight of the first field is set to *θ*_1_, the position weight of the second field is set to *θ*_2_,…, and the position weight of the *μ-*th field is set to *θ*_*μ*_. The position weight of the keyword appearing in the *j*-th field is defined as shown in the following formula:

(19) $$\begin{array}{c} W_{wz}\left(j\right)=\frac{\theta_{j}}{\sum_{i=1}^{\mu }\theta_{i}},\;i,j\in \left[1,\mu \right]. \end{array}$$

Among them, *W*_wz_(*j*) is the position weight of the keyword appearing in the *j*-th field, *θ*_*j*_ is the position weight of the *j*-th field, and ∑_*i*=1_^*μ*^*θ*_*i*_ is the sum of the position weights of all fields. Meanwhile, *i* is the summation variable, *j* is the *j*-th field, and *μ* is the number of fields. The obtained weight values *W*_*cs*_ and *W*_*wz*_ are utilized to calculate a recording vector, which is denoted by *T*, as shown in formulae (4)–(9).

(20) $$\begin{array}{c} T_{l}=\sum_{p=1}^{\mu }\left[W_{cs}\left(l,p\right)\cdot W_{wz}\left(p\right)\right]\\ =\sum_{p=1}^{\mu }\left(\frac{\lambda_{x_{I}f_{p}}}{\sum_{j=1}^{\mu }\lambda_{x_{J}f_{j}}}\cdot \frac{\theta_{p}}{\sum_{i=1}^{\mu }\theta_{i}}\right),\;l\in \left[1,n\right]\;j,p\in \left[1,\mu \right]. \end{array}$$

The parameters in formula (20) are expressed as follows: *T*_*l*_ represents the lth value of the record vector, W_cs_(*l*,*p*) represents the weight of the number of occurrences of the *l*-th keyword in the *p*-th field, and *W*_wz_(*p*) represents the position weight of the *p*-th field. At the same time, *λ*_*xlfp*_ is the number of times the *l*-th keyword appears in the *p*-th field, ∑_*j*=1_^*μ*^*λ*_*x*_*J*_*f*_*j*__ is the number of times the lth keyword appears in all fields, and *θ*_*p*_ is the position weight of the *p*-th field. ∑_*j*=1_^*μ*^*θ*_*j*_ is the sum of the position weights of all fields, *x*_*l*_ is the *l*-th keyword, *f*_*p*_ is the *p*-th field, and *μ* is the number of fields. *n* is the number of keywords.

We use the obtained “user vector” and “record vector” to find the “query value,” which is represented by *D*_*k*_, as shown in the following formula:

(21) $$\begin{array}{c} D_{k}=\sum_{l=1}^{n}Q_{l}\cdot T_{l}\\ ={\left(\begin{array}{c} Q_{1}\\ Q_{2}\\ \vdots \\ Q_{n} \end{array}\right)}^{T}\cdot \left(\begin{array}{c} T_{1}\\ T_{2}\\ \vdots \\ T_{n} \end{array}\right),\;l\in \left[1,n\right]. \end{array}$$

Among them, *Q*_*l*_ represents the *l*-th query vector, *T*_*l*_ represents the *l*-th record vector, and *k* is the number of records found. At the same time, *l* is the *l*-th vector, and *n* is the number of keywords.

## 4. Construction of Digital Management Model of Personnel Archives Information

**Figure 4:**
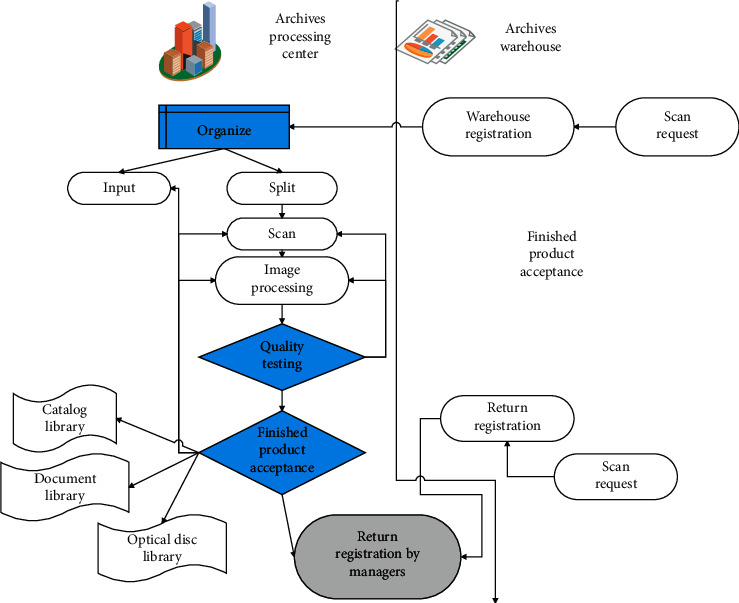
Personnel archives digital processing system.

There are two types of information resources that need to be dealt with in the digital archives project. One is the digitization of existing physical archives, and the other is the integration of existing information resources. The digitization of physical archives requires a lot of human and material investment and is a key link in the implementation of digital archives projects. Due to the complex formation and source channels of physical archives, the digital processing that can be carried out is also extremely different. There are some full-text retrievals that can be achieved by using OCR software, but due to the large amount of manual intervention, the actual feasibility is not high. Moreover, more entity archives of archives types can only be processed by means of graphic scanning supplemented by manual addition of keywords and summaries of important documents. In addition, this step is the most time-consuming link in the construction of the digital archives system. The digital processing system for personnel archives proposed in this paper is shown in Figure 4.

**Figure 5:**
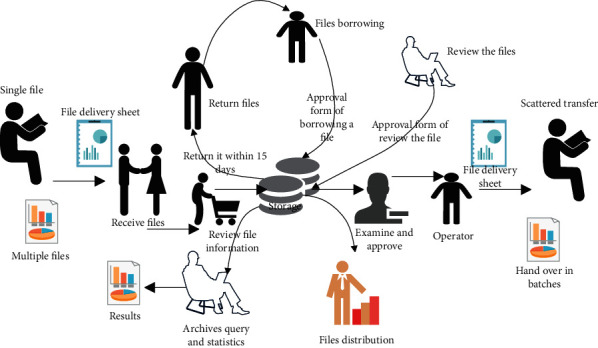
Flowchart of archives management.

The business flow chart of archives management is shown in Figure 5.

**Figure 6:**
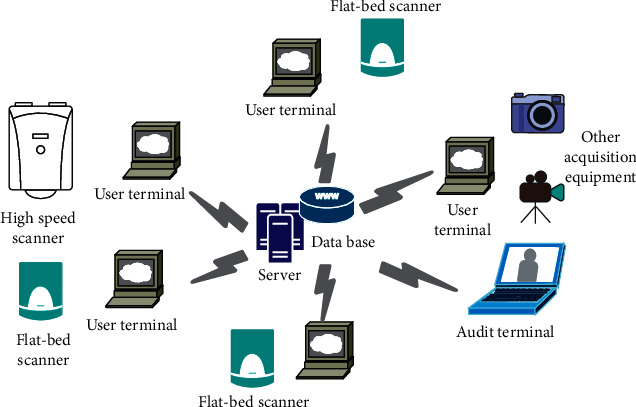
Schematic diagram of digital collection of archives.

Digital collection mainly completes the functions of digital collection and connection of paper materials. In the collection process, multiple people and multiple clients can be used to collect multiple volumes of archives and materials at the same time to achieve streamlined operations, as shown in Figure 6.

**Figure 7:**
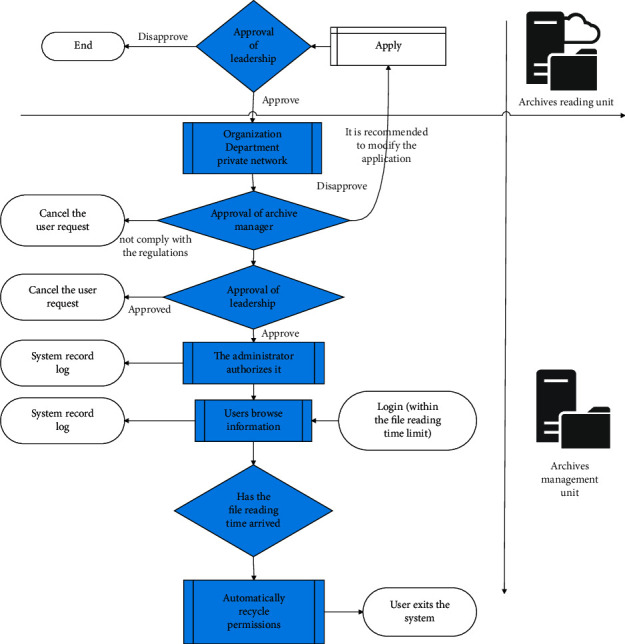
Flowchart of online viewing of personnel archives.

The review process is shown in Figure 7.

**Figure 8:**
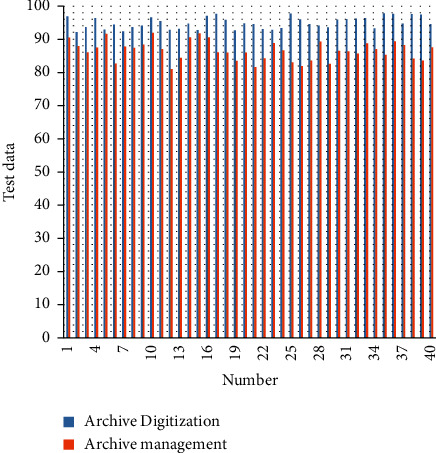
Statistical diagram of experimental data.

Based on the above research, this paper combines the concept of Industry 4.0 to verify the effect of the digital management system of personnel archives information and evaluates the digital processing of personnel archives and the effect of digital management, and the results shown in Table 1 and Figure 8 are obtained.

From the above experimental analysis, it can be seen that the digital processing and digital management platform of personnel archives proposed in this paper has a good effect of digital processing and digital management of personnel archives.

## 5. Conclusion

The digital construction of personnel archives is based on modern technology, especially computer technology and information technology. It digitalizes the traditional paper archives into digital information that can be stored on hard disks and networks. Through the processing of database technology, the scattered personal archives are transformed into a systematic and structured personal archives database. Moreover, through the digital archives database, the collection, arrangement, classification, and application of materials can be realized, and with the support of network technology, it can be transmitted through the network to realize the sharing of information materials. Digital data is the basis for realizing digital management of archives, and digital construction is an essential step in the production of digital data. In this paper, combined with digital technology, under the guidance of the concept of Industry 4.0, the digital management model of personnel archives information is constructed. The experimental research results show that the digital management model of personnel archives proposed in this paper has certain effects.

## Figures and Tables

**Table 1:** The effect of digital processing and digital management of personnel archives.

Number	Archive digitization	Archive management
1	96.87	90.50
2	92.13	87.94
3	93.70	86.05
4	96.34	87.46
5	92.85	91.58
6	94.45	82.64
7	92.33	87.76
8	93.68	87.42
9	94.11	88.33
10	96.56	91.90
11	95.45	87.04
12	92.81	81.05
13	93.15	84.46
14	94.71	90.59
15	92.70	91.73
16	97.04	90.58
17	97.67	86.02
18	95.82	85.96
19	92.66	83.49
20	94.80	85.96
21	94.57	81.58
22	93.10	84.19
23	92.80	88.90
24	93.37	86.61
25	97.78	83.05
26	96.01	81.90
27	94.59	83.54
28	94.10	89.33
29	93.63	82.59
30	95.90	86.47
31	96.05	86.32
32	96.16	85.65
33	96.32	88.82
34	93.32	87.02
35	97.83	85.28
36	97.70	89.42
37	94.75	88.20
38	97.61	84.17
39	97.47	83.56
40	94.61	87.55

## Data Availability

The labeled dataset used to support the findings of this study are available from the corresponding author upon request.
